# Follicular lymphoma within an ovarian teratoma

**DOI:** 10.1093/omcr/omag010

**Published:** 2026-04-14

**Authors:** Franceska Tchapdeu, Neha Bhardwaj, Jay Dhanapal, John Willan

**Affiliations:** John Radcliffe Hospital, Oxford University Hospitals NHS Foundation Trust, Headley Way, Oxford, OX3 9DU, United Kingdom; John Radcliffe Hospital, Oxford University Hospitals NHS Foundation Trust, Headley Way, Oxford, OX3 9DU, United Kingdom; Wexham Park Hospital, Wexham Street, Frimley Park Hospitals NHS Foundation Trust, Wexham, SL2 4HL, United Kingdom; John Radcliffe Hospital, Oxford University Hospitals NHS Foundation Trust, Headley Way, Oxford, OX3 9DU, United Kingdom; Wexham Park Hospital, Wexham Street, Frimley Park Hospitals NHS Foundation Trust, Wexham, SL2 4HL, United Kingdom

**Keywords:** haematology, oncology, lymphoma, Teratoma

## Abstract

We report an unusual case of a 46 year old diagnosed with follicular lymphoma within an ovarian teratoma. She sought medical attention due to menorrhagia, but was otherwise well. Positron Emission Tomography (PET) scanning demonstrated stage III disease, and she was treated with combination chemotherapy to complete metabolic remission on her end of treatment PET scan.

A 46-year-old laboratory assistant presented with menorrhagia. She was otherwise entirely well, with no significant past medical history, and no regular medications. Blood tests were unremarkable. An ultrasound scan demonstrated a complex right ovarian cyst of 6.1 × 3.2 cm with hyperechoic fat, calcification and a thickened endometrium, biopsy of which revealed atypical hyperplasia. The patient underwent an uncomplicated total abdominal hysterectomy with bilateral salpingo-oophorectomy (Figure 1).

**Figure 1:**
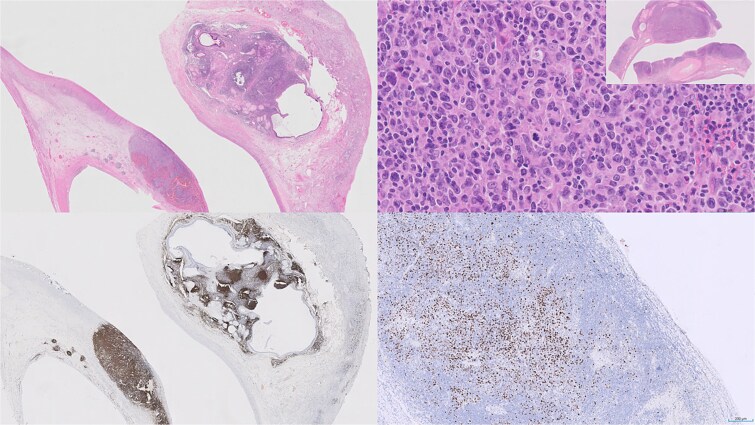
Examination revealed a solid-cystic teratomatous right ovary (top-left panel) with lymphoid infiltrate arranged in follicles. 40x magnification (top right) shows these cells to be large, centroblastic, CD20-positive (lower-left) B-cells which were also positive for CD10, Bcl-2 and Bcl-6, with preserved dendritic cell meshworks (not shown). Ki-67 proliferation index (lower-right) was 40–50% and highlighted the larger cells. 10–20% of cells were positive for C-myc, with gain of MYC in 55 of 100 cells, but no rearrangement seen. A diagnosis of follicular large B-cell lymphoma was reached as per the 5^th^ edition of the World Health Organisation classification of Haematolymphoid tumours.

Whole body positron emission tomography (PET) scanning showed increased avidity of the tonsils and submental lymph nodes. The patient was treated with 6 cycles of Rituximab, Cyclophosphamide, Doxorubicin, Vincristine and Prednisolone (R-CHOP) chemotherapy with complete metabolic resolution on an end of treatment PET scan.

Ovarian teratomas are usually benign, and are typically treated by surgical resection. They are often asymptomatic, but can cause pain or urinary symptoms. The finding of lymphoma within a teratoma is exceptionally rare, and generally such lymphomas are high-grade, typically diffuse large B-cell lymphoma [1]. To our knowledge, only one other case of Follicular large cell lymphoma (at publication this entity was classified as grade 3B follicular lymphoma) has previously been reported in the English literature [2], that patient being treated with surgery and systemic chemotherapy to complete metabolic remission, sustained at 23 months after diagnosis.
